# Linking brain and behavior states in Zebrafish Larvae locomotion using hidden Markov models

**DOI:** 10.1371/journal.pcbi.1013762

**Published:** 2026-01-05

**Authors:** Mattéo Dommanget-Kott, Jorge Fernandez-de-Cossio-Diaz, Monica Coraggioso, Volker Bormuth, Rémi Monasson, Georges Debrégeas, Simona Cocco

**Affiliations:** 1 Institut de Biologie Paris-Seine (IBPS), Laboratoire Jean Perrin, Sorbonne Université, CNRS, Paris, France; 2 Université Paris Cité, Paris, France; 3 Laboratory of Physics of the École Normale Supérieure, CNRS UMRPSL Research, Sorbonne Université, Université Paris Cité, Paris, France; 4 Université Paris-Saclay, CNRS, CEA, Institut de Physique Théorique, Gif-sur-Yvette, France; University of California Santa Barbara, UNITED STATES OF AMERICA

## Abstract

Understanding how collective neuronal activity in the brain orchestrates behavior is a central question in integrative neuroscience. Addressing this question requires models that can offer a unified interpretation of multimodal data. In this study, we jointly examine video-recordings of zebrafish larvae freely exploring their environment and calcium imaging of the Anterior Rhombencephalic Turning Region (ARTR) circuit, which is known to control swimming orientation, recorded *in vivo* under tethered conditions. We show that both behavioral and neural data can be accurately modeled using a Hidden Markov Model (HMM) with three hidden states. In the context of behavior, the hidden states correspond to leftward, rightward, and forward swimming. The HMM robustly captures the key statistical features of the swimming motion, including bout-type persistence and its dependence on bath temperature, while also revealing inter-individual phenotypic variability. For neural data, the three states are found to correspond to left- and right-lateral activation of the ARTR circuit, known to govern the selection of left vs. right reorientation, and a balanced state, which likely corresponds to the behavioral forward state. To further unify the two analyses, we exploit the generative nature of the HMM, using neural sequences to generate synthetic swimming trajectories, whose statistical properties are similar to the behavioral data. Overall, this work demonstrates how state-space models can be used to link neuronal and behavioral data, providing insights into the mechanisms of self-generated action.

## Author summary

How does spontaneous brain activity give rise to self-initiated actions? Using larval zebrafish, we combine two kinds of data: freely-swimming trajectories and neuronal recordings of a small hindbrain circuit (ARTR) known to control swim orientation. We train three-state Hidden Markov Models (HMM) separately on both datasets, enabling us to link the alphabet of actions - forward, leftward and rightward swim bouts - with stereotypical ARTR states. This parallel modeling sheds a new light on how the ARTR endogenous dynamics constrain fish swimming statistics, and its dependence on water temperature. We further show that the neuronal HMM can be used to generate naturalistic swimming sequences, while the behavioral model captures idiosyncratic features of individual fish. Overall, this work provides a generic approach to link neuronal and behavioral data.

## 1 Introduction

Animal behavior unfolds as a structured sequence of stereotyped motor actions, much like language. Understanding behavior thus requires identifying the vocabulary, *i.e.* the elementary behavioral units, and characterizing the corresponding grammar, *i.e.* their relative organization in time [1]. Uncovering this underlying structure is non-trivial. Over the last decade, numerous approaches have been proposed, building on the rapid development of data-driven computational methods. State-space models, in particular, appear to be well adapted, as they offer an unsupervised approach to sparse high-dimensional data into discrete states, while simultaneously unveiling their temporal structure. These include various implementations of Hidden Markov Models (HMMs) [2–6] and other statistical models [7–9].

Since behavior is driven by the brain activity, one expects the behavioral structure to be reflected in the spontaneous brain dynamics in the form of a sequence of discrete "brain states" - defined as metastable patterns of activity [10]. Neural activity can, as behavioral data, be parsed to uncover neural states and their temporal sequences [11–14]. In general, however, behavioral or neuronal data are analyzed separately, as these experiments are typically conducted independently, limiting our ability to bridge the two processes. In contrast, a common modeling framework, when applied to both behavior and spontaneous neural activity, could help uncover a shared organizational structure linking self-generated neuronal dynamics and behavior.

Our model behavior is the spontaneous navigation of zebrafish larvae (see [9,15–17]), which consists of discrete swimming bouts lasting ∼100 ms and triggered at ∼1−2 Hz. In previous studies the categorization of bouts was carried out independently of the examination of their temporal organization. In [18], the authors used PCA-based automatic segmentation to distinguish 13 different bout types, a number that they found sufficient to encompass the entire behavioral repertoire of the animal, including hunting, escape, social behavior, etc. However, in more constrained conditions, when the fish merely explore its environment [19–25], a simple 3-state categorization is sufficient to describe their trajectories. In this case, the bouts are labeled as either forward, left-turn or right-turn based on the value of bout-induced body reorientation. The selection of these various bout types depends on sensory cues, resulting in the animal’s capacity to ascend light [19,22] or temperature [24,26–28] gradients.

Importantly, the neural circuit that controls the orientation of bouts has been identified as the Anterior Rhombencephalic Turning Region (ARTR), a bilaterally distributed circuit located in the anterior hindbrain. Using combined calcium imaging and motor nerve recordings, it was shown that the triggering of leftward and rightward bouts are correlated with increased activity on the corresponding side of the ARTR [20].

To characterize the behavioral and neural activities and their possible relationship, we hereafter re-analyze video recordings of freely swimming animals and ARTR recordings, performed at various water temperature, using Hidden Markov Models (HMM). First, we show that for the behavioral data, this approach provides an unbiased and therefore more consistent method of bout-type labeling compared to simple thresholding techniques as used in earlier studies. We further use the HMM inferred parameters to demonstrate and quantify inter-individual variability in exploratory kinematics. We then apply a 3-states HMM to the ARTR recordings performed in paralyzed tethered fish, leading to the comparison between the behavioral and neural HMMs. Finally we generate synthetic neuronal-based swimming sequences and compare the statistical structure of these synthetic trajectories with real ones to assess the consistency of the results across both behavioral and neural data.

## 2 Results

### 2.1 Data

**Fig 1:**
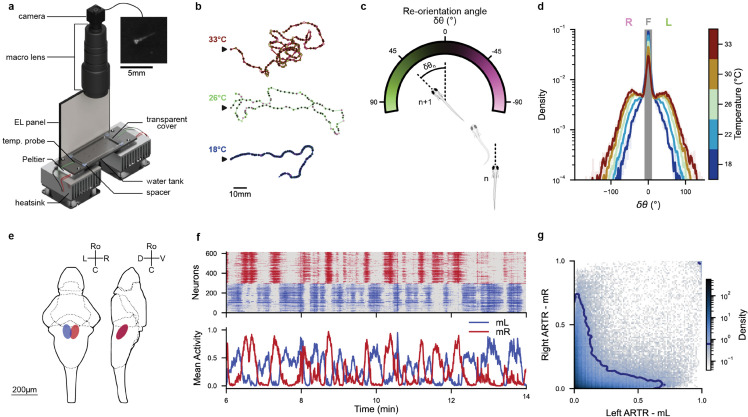
Behavioral and neuronal datasets: **(a)** Overview of the experimental setup: Zebrafish larvae are free to move in a tank that is kept at a desired constant temperature by a Peltier module. An imaging system records images of the fish from above at a rate of 25 frames per second. The upper right panel provides a close-up view of a larva in a raw image. Adapted from [24]. **(b)** Example trajectories of zebrafish larvae in 2D space at various temperatures. Each point represents a swim bout, with the color indicating the corresponding re-orientation angle defined in panel c. The trajectories’ starting points are denoted by black arrows. **(c)** Convention used for the reorientation angle ($\delta \theta_{n}$) between two consecutive swim bouts (*n* and n+1). **(d)** Distribution of re-orientation angles ($\delta \theta_{n}$) for each ambient temperature. The grayed-out area corresponds to the re-orientation angles classified as forward bouts by a thresholds at $\pm 10^{\circ }$. **(e)** Diagram of the *Anterior Rhombencephalic Turning Region* (ARTR) in larval zebrafish. Adapted from [29]. **(f)** Example ARTR activity at 22°C. Top : Raster plot of neurons located in the left and right ARTR (blue and red respectively). Bottom : Mean activity *m*_*L*_ and *m*_*R*_ of neurons in the left and right ARTR. **(g)** Mean activities $\left(m_{L},m_{R}\right)$ of the ARTR for all recordings in the dataset. The blue contour line represents 90% of the joint distribution.

The behavioral data used in the present article comes from a publication that examined the kinematic of free exploration in zebrafish larvae [24]. The experimental design (Fig 1a) enables recording the trajectories of multiple freely swimming larvae aged 5-7 days at temperatures of 18°C, 22°C, 26°C, 30°C, and 33°C. At each temperature, the trajectories of multiple fish are combined into a single dataset, and a set of kinematic parameters is extracted at each bout *n*, such as the angular change $\delta \theta_{n}$ in heading direction, the time elapsed since the previous bout and the traveled distance (see Material and methods Sect 4.1). Water temperature was found to systematically impact the statistics of navigation, leading to qualitatively different trajectories as illustrated in Fig 1b. As the temperature increases, trajectories tend to become more winding and erratic. We have also re-analyzed a second dataset of long-trajectories for 18 fish tracked individually for over two hours at 26°C, in order to assess inter-individual variability (see Material and methods Sect 4.1).

The neural data comes from another publication in which the spontaneous activity of the *Anterior Rhombencephalic Turning Region* (ARTR) [29] (Fig 1e) was recorded from 5-7 days old immobilized larvae expressing the calcium indicator GCaMP6f, using light-sheet functional imaging. Several neural recordings (3-10) for each one of the five temperatures (from 18°C to 33°C (Fig 1b)) were analyzed. The fluorescence signal of each neuron was further deconvolved to estimate an approximate spike train (see Material and methods Sect 4.2).

### 2.2 Modeling of behavior

#### 2.2.1 Markov models.

The distribution of reorientation angles after each bout, shown in Fig 1d, appears to be trimodal, suggesting a classification of the bouts in 3 types: forward (*F*), left-turn (*L*) and right-turn (*R*). In practice, this categorization is generally carried out by thresholding the distribution of re-orientation angles. Denoting the state of swim bout *n* by *s*_*n*_ we have:

$$s_{n}=\left\{ \begin{array}{cc} R, & \text{if }\delta \theta_{n}<-\delta \theta_{0}\\ F, & \text{if }-\delta \theta_{0}<\delta \theta_{n}<+\delta \theta_{0}\\ L, & \text{if }\delta \theta_{n}>+\delta \theta_{0} \end{array}\right.$$ (1)

**Fig 2:**
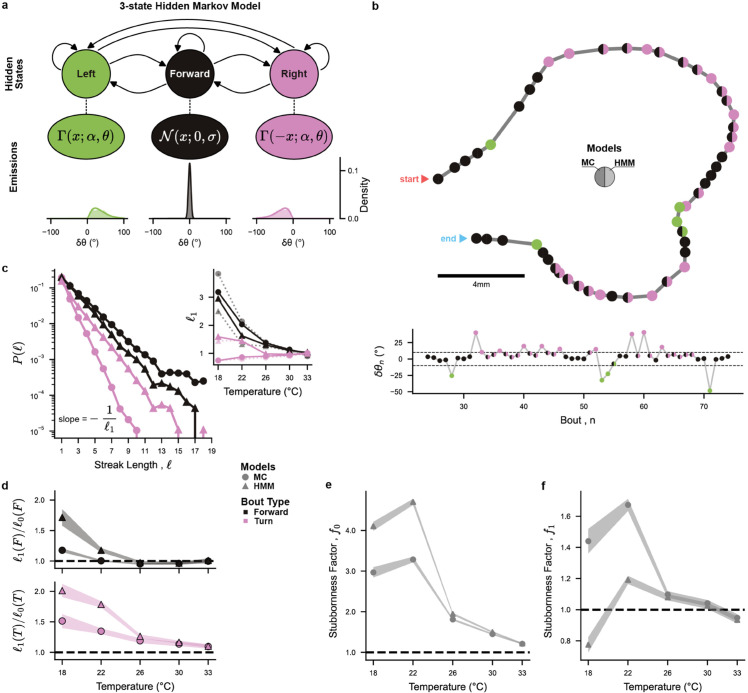
3-state Markov Chain and hidden Markov models - Stronger persistence emerges from better labeling: **(a)** Diagram of the 3-state Hidden Markov Model (HMM) with normal emissions for Forward bouts, and gamma emissions for Turning bouts. Example emission distributions were taken at 26°C. **(b)** Example trajectory at 22°C. Each point represents a swim bout, with the left color for the threshold labeling (Markov Chain model), and the right color for the HMM labeling using the Viterbi algorithm. Top: 2D trajectory. Bottom: reorientation angle $\delta \theta_{n}$ for this trajectory, with the threshold $\delta \theta_{0}=\pm 10^{\circ }$ as a dashed line. **(c)** Probability P(ℓ) of observing a streak of ℓ consecutive forward bouts (black) or same-direction turning bouts (pink), for MC (circles) and HMM (triangles), at 22°C. Inset: Exponential decay characteristic length ($\ell_{1}$, solid lines), and theoretical persistence length computed from the transition matrix ($\ell_{1}(s)=-1/\ln P(s\to s)$, dashed lines). **(d)** Ratio of persistence length $\ell_{1}/\ell_{0}$ (observed vs. no-memory null model) vs. temperature, for Forward (*s* = *F*, black) and turning (s∈L,R, pink) bouts. **(e)** *stubbornness* factor at *q* = 0 intermediary Forward bouts, $f_{0}=\frac{P(L\to L)\;+\;P(R\to R)}{P(L\to R)\;+\;P(R\to L)}$. **(f)** *stubbornness* factor at *q* = 1 intermediary Forward bouts, $f_{1}=\frac{P(L\to F\to L)\;+\;P(R\to F\to R)}{P(L\to F\to R)\;+\;P(R\to F\to L)}$. **(e-f)** Shaded bands represent the estimated errors from aggregated fish data (see Materials and methods 4.5).

The use of the same threshold (in absolute value) to detect left and right turns relies on the hypothesis that zebrafish larvae, as a group, have no preferred direction (*a.k.a.* non-handedness). As the exact value of $\delta \theta_{0}$ has minimal qualitative impact on the results of the Markov Chains, we adopt the same value $\delta \theta_{0}=10^{\circ }$ as in [24]; notice that $\delta \theta_{0}$ is the same across all temperatures to avoid introducing ad hoc, temperature-dependent biases. An example of the classification of states along a swimming trajectory is presented in Fig 2b.

Once the bout types are identified, we define a dynamical model for the trajectories $\ldots \to s_{n-1}\to s_{n}\to s_{n+1}\to ...$ using a three-state Markov Chain (MC). Informally, the sequence of states (associated with the 3 different bout types) is described by the probabilistic automaton in S3 Fig a. In this model, after each bout *n*, a new state *s*_*n*+1_ is drawn randomly, conditioned only on *s*_*n*_ (and not on previous states). The transition probabilities between states, $P(s=s_{n}\to s^{\prime }=s_{n+1})$, are estimated by counting the numbers # of occurrences of the transitions $s\to s^{\prime }$ along the trajectories:

$$P(s\to s^{\prime })=\frac{\#(s\to s^{\prime })}{\#(s\to F)+\#(s\to L)+\#(s\to R)}$$ (2)

with $s,s^{\prime }\in \{F,L,R\}$.

The top right eigenvector of the 3×3 transition matrix gives access to the stationary probabilities *P*(*s*) of the 3 states. As a self-consistency check, we confirmed that this stationary distribution matches the empirical fraction of threshold-labeled states measured in the same dataset, with a maximum absolute difference <0.003 for every bout type and temperature.

#### 2.2.2 Hidden Markov model.

We then turn to an alternative categorization method, where states are inferred rather than *a priori* assigned. To do so, we consider a three-state Hidden Markov Model (HMM), see Fig 2a. Unlike MC, HMM makes a clear distinction between the observations (here the reorientation angles $\delta \theta_{n}$ treated as ‘symbols’) and the states of the system (here *s*_*n*_, which are not directly accessible from the knowledge of $\delta \theta_{n}$, in contradiction with the key assumption underlying MC). The HMM is defined by:

- The transition probabilities $P(s\to s^{\prime })$ between the hidden states.
- The emission probabilities, E(δθ|s), relate the observations δθ to the hidden states *s*. For the forward state, we choose normally distributed reorientation angle emission distributions, centered in zero: E(δθ|F)=𝒩(δθ;0,σ). For turn states, we use Gamma distributed reorientation angles, with a positive or negative sign according to whether the state is Left or Right: $E(\delta \theta |L)=\Gamma (\;+\;\delta \theta ;\alpha_{L},\theta_{L})$ and $E(\delta \theta |R)=\Gamma (-\delta \theta ;\alpha_{R},\theta_{R})$, constraining $\theta_{L},\theta_{R}>0$ and $\alpha_{L},\alpha_{R}>1$. These specific functional forms (Normal and Gamma) were chosen empirically because they adequately capture the experimental turn angle distributions across all temperatures (see Material and methods Sect 4.3 for details about the validation of these emission distributions).
- A probability distribution for the initial state at the beginning of a trajectory.

We consider a symmetric HMM, where left-right directions are equivalent. This is done in practice by enforcing the following constraints over the transition probabilities,


P(F→L)=P(F→R) ,

P(L→L)=P(R→R) ,

P(L→R)=P(R→L) ,

P(L→F)=P(R→F) ,

and $\theta_{L}=\theta_{R},\alpha_{L}=\alpha_{R}$ for the emission distributions. We have checked that relaxing these constraints on the transition matrix leads to equivalent results, see S9 Fig and S10 Fig, while maintaining them leads to faster training convergence.

We train both types of HMM models using Expectation Maximization (Baum-Welch algorithm) aggregating all trajectories at a given temperature in the first dataset, and for each individual fish in the long-trajectory data. We employ a customized Julia [30,31] implementation (available at https://github.com/ZebrafishHMM2023/ZebrafishHMM2023.jl).

Results show that the inferred parameters for the unconstrained and symmetric models are very similar, see S9 Fig. This is expected since our experimental setup is compatible with left-right symmetry. In the following, we therefore employ symmetric HMM only, since imposing symmetry from the beginning results in faster and more robust training of the HMM. This in turn ensures that steady state bout probability is left-right symmetric (P(L)=P(R)).

### 2.3 State classification and behavioral persistence

#### 2.3.1 Statistics of bout states.

Since the Markov Chain inferred from thresholded data (MC, S3 Fig a) and the Hidden Markov Model (HMM, Fig 2a) share the same internal behavioral states, we can directly compare these two models and thus examine the impact of the labeling methods.

As illustrated with an example trajectory at 22°C in Fig 2b, MC and HMM labeling can differ significantly. MC-inferred sequences often exhibit multiple alternations between Forwards and Turns when the bouts reorientation angles are near the threshold, while for the same sequence, the HMM tends to consistently label these bouts as Turns. These differences result in a reclassification of approximately 60% of Forward bouts into Turning bouts at 22°C (S3 Fig e).

The HMM yields a relatively modest dependence of bout-type usage on temperature (see S3 Fig b). In contrast, the threshold classification method used in MC lead to a systematic and pronounced increase in the fraction of turning bouts with rising temperature. This strong temperature dependence, previously reported in [24], may have thus been overestimated, as it partly reflects the ad-hoc assumption of a fixed (temperature-independent) threshold $\delta \theta_{0}$. Conversely, the HMM approach infers a gradual widening of the forward bouts angular distribution with increasing temperature that corresponds to an increase in the effective angular threshold (see S2 Fig c–e).

#### 2.3.2 Bout streaks and persistence.

We further assessed how bout-type persistence, defined as the tendency to execute similar bouts in succession, depends on the chosen classification model. We start by describing trajectories as a series of streaks of similar bouts (forward, leftward or rightward), and then characterize the streak length distribution. For all bout types and models, the probability of observing a streak of ℓ consecutive bouts of the same type decays exponentially, $P(\ell )\propto e^{-\ell /\ell_{1}}$, with $\ell_{1}$ defining the characteristic streak length (Fig 2c). For turning bouts, we found $\ell_{1}^{\text{HMM}}\approx 1.4\text{ bouts}$ while $\ell_{1}^{\text{MC}}\approx 0.9\text{ bouts}$ at $22^{\circ }C$. Compared to MC, HMM-based labeling thus yield much longer turning streaks. In contrast, we find no significant difference in characteristic forward-streak length, with MC having slightly longer streaks than HMM. As temperature increases, we observe for both models that the characteristic streak length decreases (particularly for forward bouts, see Fig 1b).

Within the Markov or Hidden Markov Model frameworks, the average length $\ell_{1}(s)$ of a streak of bouts of type *s* is related to the probability P(s→s) of remaining in the same state through the simple relation $\ell_{1}(s)=-1/\ln P(s\to s)$. To distinguish the effects on bout-type persistence due to the presence of memory from the mere consequences of single-state frequencies, we introduce a null model in which the transition probabilities are simply given by these frequencies, *i.e.* $P(s\to s^{\prime })=P(s^{\prime })$. In this null model without any memory, the average length of type-*s* bouts is simply $\ell_{0}(s)=-1/\ln P(s)$. The ratio $\ell_{1}(s)/\ell_{0}(s)$ is an estimator of the (relative) contribution of behavioral memory to bout-type persistence.

Results are shown in Fig 2d for the Markov (MC) and Hidden Markov (HMM) Models. The MC and HMM methods yield comparable outcomes for turning bouts at low temperature. However, HMM-based analysis further reveals a persistence for forward bouts at lower temperatures (Fig 2d), while this effect is absent in the MC model. This absence of forward persistence was previously reported in [22], and we hypothesize that it is due to the mis-labeling associated with the threshold method. Interestingly, such persistence effects vanish at higher temperatures, where the transition matrix becomes uniform (S3 Fig c,d), and all bouts become equiprobable (P(F)≈P(L)≈P(R)). One thus expect more erratic trajectories at higher temperatures, which is consistent with our observations (see Fig 1b).

#### 2.3.3 Consistency of the MC and HMM descriptions of behavior.

Taken together, the results above suggest that the Hidden Markov Model better captures persistence in reorientation by labeling bouts with small reorientation angles based on context. This leads to a more flexible and thus stable classification than the thresholding method. However, given the absence of a ground truth, it remains unclear whether the labeling produced by the Hidden Markov Models is more accurate than the one produced by the standard threshold-based approaches.

One way to address this question is to examine to what extent each of these methods are self-consistent, i.e. guarantees that the inferred labeled sequences are truly markovian such that the bout type at a given time only depends on the type of the preceding bout. It has been previously noted that the thresholding methods lead to significant non-markovianity. In particular, in a transition $T_{1}\to F\to T_{2}$ with $T_{1},T_{2}\in \{L,R\}$, the two turning bouts tend to have the same orientation ($T_{1}=T_{2}$). This means that the memory of orientation *T*_1_ is maintained during the forward bout, in violation of the Markovian assumption. This observation led to propose a 4-state Markov system comprising two independent Markov chains, independently controlling the forward-turn bout transitions, and directional left-right bout transitions (see S4 Fig b for a diagram of this 4-state model) [22,24].

Given that our 3-state Hidden Markov Model (HMM) re-labels numerous Forward bouts as Turn bouts, we ask whether this new classification might alleviate this non-Markovianity issue, such that the ad hoc 4-state model might no longer be needed. We thus propose a new test of Markovian violation specifically designed for our use case, that we apply to both the HMM and MC models.

We introduce the *stubbornness factor f*_*q*_ to empirically assess the tendency of larvae to retain their orientation after a sequence of *q* intermediary forward bouts (S4 Fig b, Materials and methods Sect 4.5):

$$f_{q}=\frac{\sum_{T_{1}=T_{2}}P(T_{1}\to F^{q}\to T_{2})}{\sum_{T_{1}\neq T_{2}}P(T_{1}\to F^{q}\to T_{2})}$$ (3)

with $T_{1},T_{2}\in \{L,R\}$ and $F^{q}=\underset{q}{\underbrace{F\to F\to \cdots \to F}}$.

Owing to the loss of orientational memory after a forward bout, a non-handed 3-state Markovian model should have *f*_*q*_ = 1 for q≥1 (Materials and methods Sect 4.6). On the other hand, *f*_*q* = 0_ is a measurement of directional persistence during uninterrupted sequences of turning bouts.

We found that most of the memory effects captured by the HMM occur at *q* = 0, and that the *stubbornness* reaches $f_{q}\approx 1$ for q≥1, suggesting that the HMM-inferred bout sequences are quasi-Markovian. In comparison, and for lower temperatures, the thresholded MC classification displays lower persistence at *q* = 0 but higher *stubbornness* at *q* = 1 as seen in Fig 2e–2f (and less significantly at *q* = 2, see S4 Fig d). This suggests that the thresholded labeling leads to Markov violation primarily due to the mislabeling of turn bouts as forward bouts during turning streaks, as anticipated in the previous section and illustrated in Fig 2b. As this *stubbornness* is mostly significant at *q* = 1, we expect that most mislabelings are one-off errors.

In summary, previous works using an ad hoc threshold to classify bouts had dismissed 3-state Markov models because the resulting sequences were non-markovian. We found that by using an unsupervised method to simultaneously label the data and infer a Markov Model, we could unveil previously underestimated memory effects in zebrafish reorientation statistics. These results suggest that the HMM labeling is more markovian, making it a more coherent model than MC. This is probably due to a reclassification of forward bouts as turns during sequences of small reorientations.

### 2.4 Behavioral phenotyping from long individual fish trajectories

As HMM provides an unbiased quantification of the behavior, we now ask whether the approach is accurate enough to detect behavioral differences between specimen (inter-individual variability) and whether it can enable the unambiguous identification of each animal.

In the preceding sections, the dataset used to infer the models comprised trajectories from multiple fish, as the different individuals swimming together during a given assay could not be distinguished. To address the question of individuality, we used additional experiments reported in [24], in which individual fish were tracked at 26°C (see Materials and methods 4.1). A total of 18 fish were recorded for over 2 hours. We first trained HMMs on such long trajectories (the “global HMM”), by enforcing left-right symmetry on the transition rates as for the aggregated fish data. For comparison, we show in S10 Fig the results when we trained HMMs by relaxing the left-right symmetry on the transition matrix. The resulting transition matrices are close to be symmetric, even if slight deviations may be found for some fish.

**Fig 3:**
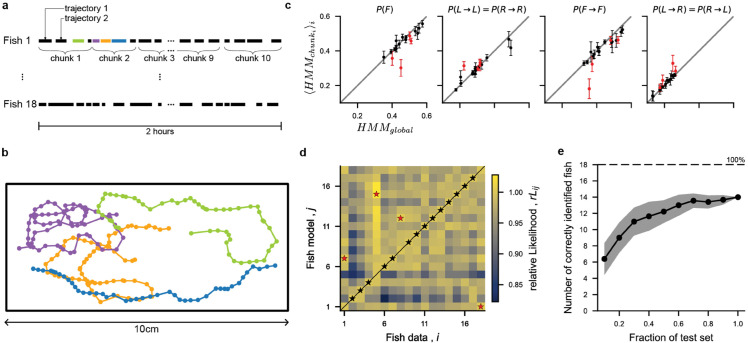
Fish identification from long trajectories: **(a)** Dataset : 18 fish recorded individually for 2-hour sessions. Each session is split into 10 chunks (mean = 9.5 ± 0.5 trajectories per chunk). **(b)** Example trajectories for fish 1. **(c)** HMM parameters inferred from all the trajectories of a fish (referred to as global) vs. inferred per-chunk. Only four HMM parameters are shown for clarity (see S5 Fig for all parameters). Each dot represents a fish, and the error bars correspond to the standard error of the mean. Points labeled in red correspond to fish misidentified in panel d. **(d)** Confusion matrix of the relative likelihood $rL_{i,j}=\frac{L(data_{i}|model_{j})}{L(data_{i}|model_{i})}$ of data coming from fish i and HMM trained on fish j. The fish identity most likely according to each model is indicated with a star (black : correctly identified fish, red : misidentified). **(e)** Average number of correctly identified fish when a fraction *f* of the test data is used for identification. Shaded band : standard deviation across 100 trials. In each trial, the trajectories of each fish were randomly split into train and test sets (50%).

We then split the 2h-long recorded sequence of each individual fish into smaller periods (chunks) of ≈12 minutes each, and trained an HMM on each of these chunks (see diagram in Fig 3a–3b).

For each fish, the parameters of these HMMs exhibit significant variability (as shown by the vertical error bars in Fig 3c). This variability between the different chunks reflects both intra-individual (temporal) variability and, to a lesser extent, inference uncertainty due to the limited sampling of the HMM (see S5 Fig). We stress that small deviations from symmetry (*e.g.* by relaxing left–right symmetry, see S10 Fig) would make individual fish even easier to recognize. However, since chunk trajectories are shorter and thus more sensitive to symmetry-breaking fluctuations, imposing left–right symmetry also reduces susceptibility to such errors. Fig 3c compares selected parameters of the global HMM for each fish, against the average parameters over several HMMs trained on the chunk trajectories (see S5 Fig for all parameters). There is a clear trend between the global HMM and the average behavior of the chunk HMMs. Therefore, although a fish exhibits variability during a long sequence of bouts, the variability between distinct fish is larger.

These results suggest that the HMM models can be used to distinguish different fish from observations of their bout sequences. To test this hypothesis, we split the trajectories of each fish into a training and a withheld test set. After training the HMM on the train set for a particular fish, we computed the likelihood of all fish trajectories in the test set, and compared them. For 14 out of the 18 fish, the test set that yield the maximum likelihood rightly identifies the fish used to train the HMM (Fig 3d). This finding suggests that the HMM captures behavioral parameters which are distinctive enough to discriminate between different fish. Given the large variability exhibited by a single fish, one expects this discriminative ability to increase with the duration of the training sequences. To quantify this, we further evaluated the likelihoods of subsets of the test fish trajectories, and recorded the number of times that the maximum likelihood HMM corresponded to the correct fish (Fig 3e). Even when withholding 80% of the sequence, we were able to correctly identify 10 out of the 18 fish. These results suggest that individual fish exhibit variable but distinctive behavior which can be captured by the HMM.

### 2.5 Modeling of neural data

The selection of turning bouts orientation in zebrafish is known to be controlled by a small bilaterally distributed circuit in the anterior hindbrain, called *Anterior Rhombencephalic Turning Region* (ARTR). This circuit displays self-sustained alternating activity between its left- and right-lateral sub-population, with a period of the order of tens of seconds (Fig 1e). The animal tends to execute left turns when the left ARTR is active while the right ARTR is inactive (and vice versa for right turns) [20].

In contrast, no specific circuit has yet been identified for the selection of turn vs forward bouts. The hypothesis that two distinct circuits are involved in bout-type selection is consistent with the 4 states Markovian model of navigation, in which two independent Markov chains drive the two selection processes. However, the 3-state Markovian model supported by the HMM analysis suggests that the same circuit (ARTR) could drive the selection of all 3 bout-types.

**Fig 4:**
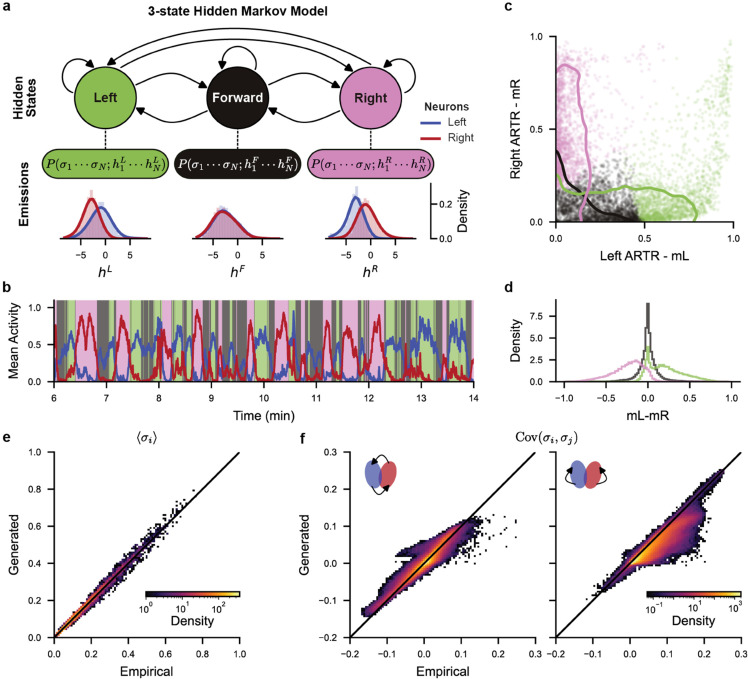
3-state Hidden Markov Model (HMM) describes ARTR neuronal statistics: **(a)** Diagram of the 3-state Hidden Markov Model (HMM) with emissions described as independent models of the ARTR neuronal population, see Eq (4). Distributions of fields $h_{i}^{s}$ are shown for all fish for neurons in the left (blue) and right (red) ARTR. **(b)** Example ARTR activity (see Fig 1f) classified by the 3-state HMM. Solid lines represent the mean activity of neurons in the left (*m*_*L*_, blue) and right (*m*_*R*_, red) ARTR. **(c)** HMM classification in $\left(m_{L},m_{R}\right)$ space. Dots : neuronal configurations from the example recording in panel b. Solid lines : 90% of the distributions for all recordings combined. **(d)** Distributions of $m_{L}-m_{R}$ per state (all recordings combined). **(e-f)** Empirical vs. HMM-generated neuronal statistics (all recordings combined). **(e)** Mean activity $\left\langle \sigma_{i}\right\rangle$ of neuron *i*. **(f)** Covariance $\text{Cov}(\sigma_{i},\sigma_{j})$ of neurons *i* and *j* on opposite sides (left plot) and on the same sides (right plot) of the ARTR.

In order to test this hypothesis, we re-analyzed the ARTR recordings reported in [29] using a 3-state HMM (Fig 4a). We posit an independent neural model for the activity of the *N* recorded neurons, yielding, for each state, the emission probability:

$$P(\sigma_{1},\sigma_{2},\ldots \sigma_{N}|s)=\prod_{i=1}^{N}\frac{e^{h_{i}^{s}\;\sigma_{i}}}{\left(1+e^{h_{i}^{s}}\right)}$$ (4)

where $\left(\sigma_{1},\sigma_{2},\ldots \sigma_{N}\right)$ is a neuronal configuration, *s* is the hidden state, and $h_{i}^{s}$ is the local field representing the effective excitability of neuron *i* in state *s*. The model thus includes 3×N parameters $h_{i}^{s}$, associated to each neuron and each hidden states. Notice that for the neural HMM, the non-handedness of the behavioral HMM is not enforced. We also performed comparisons of neural HMM with different numbers of hidden states in a cross-validation test, and found that including more than 3 hidden states results in marginal improvement, see S8 Fig.

The distribution of fields $h_{i}^{s}$ for the 3 hidden states, shown in Fig 4a, are used to assign labels to the three states (see Materials and methods 4.7). Consistent with our current understanding of the ARTR function for turn selection, the state with large values of the fields on the left and smaller values of the fields on the contralateral side is labeled "left" (and vice versa for "right"). The third state exhibits similar distributions of fields for neurons on the left and right side of the ARTR, and is labeled forward in analogy with behavior. The ARTR activity is thus modeled as a sequence of left-right-forward states.

With this classification, the forward state corresponds to a low mean neuronal activity of both the left (*m*_*L*_) and right (*m*_*R*_) sides of the ARTR, while turning states are associated with large activity on the ipsilateral side of the ARTR (left state : $m_{L}>m_{R}$, right state : $m_{L}<m_{R}$, see Fig 4b–4d).

This model accurately captures the mean activity of each neuron (Fig 4e), as well as the pairwise correlations between contralateral neurons. However, ipsilateral pairwise correlations are not as well reproduced, showing lower covariance in the generated data (Fig 4f). This mismatch presumably comes from the fact that the activities of neurons within a state are uncorrelated in our emission probabilities, while recurrent interactions in the ARTR circuit produce correlations. These would be better modeled with emission probabilities including effective interactions between neurons [29].

### 2.6 Comparison of behavior and neuronal HMMs

In the preceding sections, we demonstrated that both the reorientation behavior and the neuronal activity of the *Anterior Rhombencephalic Turning Region* (ARTR) can be effectively modeled using three-state Hidden Markov Models (HMMs). However, it remains unclear whether the three states identified in the Behavioral HMM (B-HMM) directly correspond to those inferred in the Neuronal HMM (N-HMM).

Unfortunately, there is currently no publicly available dataset offering simultaneous recordings of freely swimming larvae kinematics and neuronal activity, which would enable direct comparison of B-HMM and N-HMM states for individual bouts. Current research addressing this question largely relies on experimental paradigms where larvae are either paralyzed with electrophysiological recording of motor nerve signals (fictive swimming preparations) [20,32,33], or head-embedded with a free-moving tail (head tethered preparations) [34–37]. In fictively swimming preparations, whilst the classification of left-vs-right bouts is feasible based on the asymmetric nature of the motor command, such experiments are not, to the best of our knowledge, capable of discriminating forward-vs-turning bouts [20]. On the other hand, head tethered preparations allow forward-left-right bout classification [34,36], but typically rely on visual stimuli to elicit behavior [34–37] as the spontaneous sequence of bouts is strongly disrupted in comparison with freely swimming contexts [38]. The disruption of behavioral and neuronal dynamics caused by animal immobilization is a general problem in behavioral neuroscience, as illustrated by studies in C. *elegans* where the direct comparison of neuronal dynamics between freely-moving and immobilized worms was quantified [39].

**Fig 5:**
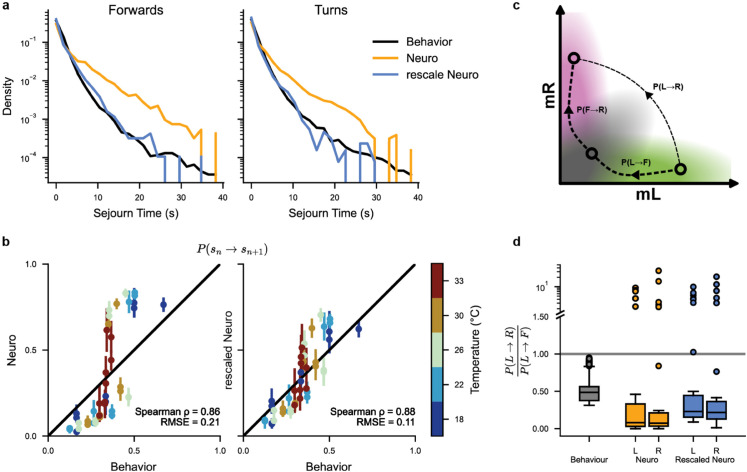
Behavior vs. Neuronal temporal structure: **(a)** Sojourn-time distribution for forward (left) and turn states (right) : behavior (black), neuronal before (orange) and after (magenta) temporal re-scaling. A single re-scaling factor is used for forward and turning states, for all temperatures, and recordings. **(b)** Behavior vs. neuronal state-transition probabilities $P(s_{n}\to s_{n\;+\;1})$ (for all state pairs and all temperatures), before (left) and after (right) temporal re-scaling. Each dot represents a single transition probability at a given temperature. For neuronal state-transition, we show the mean and standard error over all recordings. **(c)** Diagram showing two possible transition trajectories between left and right states in ARTR $\left(m_{L},m_{R}\right)$ space. Transitions through the forward state are more probable (see panel d). **(d)** Distributions of $\frac{P(L\to R)}{P(L\to F)}$ for behavior (black) and neuronal data before (orange) and after (magenta) temporal re-scaling (all temperatures combined). These distributions are shown as standard box plots (median, quartiles, and outliers beyond 1.5× the inter-quartile range from the median).

We hereafter propose to circumvent these experimental challenges by comparing the statistical structures of the reorientation sequences inferred from the two datasets presented in Sects 2.1 and 2.5. The transition probabilities $P(s_{n}\to s_{n+1})$ obtained from B-HMM and N-HMM at all recorded temperatures are shown in Fig 5b. Comparison of these transition rates require to first correct them for differences in sampling rates. Indeed, neural transition rates are computed from neuronal recordings performed at ∼6 Hz (depending on the dataset, see Materials and methods 4.2), while for behavior, the sequences are divided into swim bouts triggered at an average rate of ∼1 Hz depending on the temperature.

To bridge the gap between neuronal and behavioral datasets, one needs to estimate how the behavior is subsampled from the neuronal activity. Previous observations have shown that internal dynamics, and precisely ARTR pseudo-period, tends to be slower in head-tethered assays [29], as well as experiments comparing free and fictive swimming which showed that the fictive swimming frequency is reduced in paralyzed animals [20]. To verify this, we computed the distribution of sojourn times $\Delta t_{s}$ of all three states in both B-HMM and N-HMM, where $\Delta t_{s}=t_{k}\;-\;t_{1}$ is the duration of a sequence $\left(s_{1},...,s_{k}\right)$ of *k* consecutive states *s* observed at times $\left(t_{1},...,t_{k}\right)$. We found the neuronal sojourn times to be significantly longer than the behavioral sojourn times (Fig 5a). The optimal temporal scaling factor *f*_*N*/*B*_ for which the distribution of neuronal sojourn times matches the distribution of behavioral sojourn times (see Materials and methods 4.8) was $f_{N/B}\approx 0.44$. Interestingly, this value appears to be consistent with findings from [20], which reported the mean interbout interval for fictive swimming to be 0.41 times slower than for freely swimming.

Using this temporal re-scaling factor, we find that the transition probabilities $P(s_{n}\to s_{n+1})$ for behavior and ARTR models are similar (RMSE=0.1, see Fig 5b), indicating that the behavioral and neuronal state sequences share similar underlying structures. This is remarkable as the number and meaning of the neuronal internal states were not *a priori* fixed, but entirely assigned by N-HMM after training.

This result supports our hypothesis that the ARTR not only governs the selection between rightward and leftward turning bouts, but also controls the bout-type selection, forward *vs* turn. To test this claim further, we analyzed in more detail the statistics of trajectories in the bout space inferred from the ARTR dynamics and from behavioral data. We specifically examined the bout sequences leading to a change in orientation, such as transitions from *L* to *R* and vice-versa. Such orientational switches can be either direct, e.g. L→R, or may include an intermediate forward bout, L→F→R (Fig 5c). Using the ARTR signal, we found that the second path is strongly favored as evidenced by the the fact that $\frac{P(L\to R)}{P(L\to F)}<<1$. A comparable value of this ratio is observed in the behavioral data (Fig 5d), indicating that fish indeed tend to execute a forward bout when changing orientation. This statistical bias would be difficult to understand under the standard model that posits the existence of independent neural circuits governing orientation and bout-type selection, respectively. In contrast, in our model, it emerges naturally from the phase space structure of the ARTR dynamics as shown in Fig 4c and Fig 5c. The L-Shaped distribution of $\left\{m_{l},m_{R}\right\}$ constrains the Left-to-right (or Right-to-Left) trajectories to pass through a symmetrical, low activity state, thus favoring intermediate forward bouts.

### 2.7 Generation of synthetic behavior with the neural model

So far we have compared neuronal and behavioral data by examining only the short-scale statistical structures of the HMM-inferred state sequences. We now use the N-HMM model to identify state sequences from activity recordings, then generate long synthetic trajectories and compare their statistics with those of freely swimming fish. This approach allows us to assess whether the N-HMM, when combined with appropriate scaling and behavioral parameters, can reproduce the complex statistical properties of exploration at various temperatures.

#### 2.7.1 Generation of trajectories from neural recordings.

Consider a neural activity recording. Following Sect 2.6, we hypothesize that the internal states obtained by parsing the activity recording with the Neural HMM (N-HMM) match the behavioral internal states, after proper temporal rescaling. Therefore, we expect that it should be possible to generate artificial swim trajectories from these neural states.

**Fig 6:**
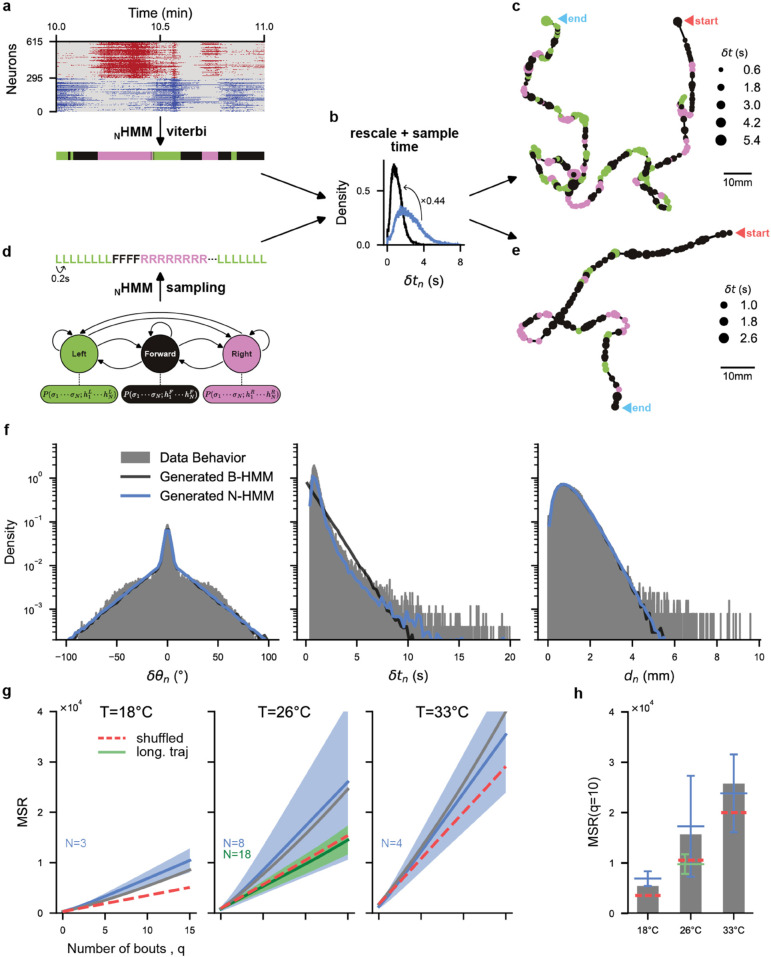
Generative ability of HMM models and trajectory reconstruction from neural HMMs: **(a,b,c)** Pipeline to convert neuronal activity to swim trajectory. **(a)** ARTR activity is first converted to the most likely sequence of forward/left/right hidden states using the Viterbi algorithm on the Neuronal Hidden Markov Models (N-HMM). (Example empirical ARTR activity at 26°C). **(b)** Time is then re-scaled using the scaling factor identified in Fig 5, and sampled based on the empirical distribution of inter-bout intervals $\delta t_{n}$. Bout-angle sequences are then sampled based on the Behavioral Hidden Markov Models (B-HMM). **(c)** Swim trajectories are constructed for each bout sequence by sampling the bout distances *d*_*n*_ from the empirical distribution. An example trajectory generated from the empirical neuronal activity at 26°C is shown. Point color corresponds to bout type (left, right, forward), and point size corresponds to inter-bout interval. **(d,b,e)** Pipeline to convert N-HMM-generated state sequences to swim trajectories. **(d)** The N-HMM is first sampled to generate a sequence of forward/left/right internal states. The sequence is then temporally rescaled and sampled as before (see panel b). **(e)** Swim trajectories are constructed as before (see panel c). An example N-HMM-generated trajectory at 26°C is shown. **(f)** Distributions of reorientation angles $\delta \theta_{n}$, inter-bout intervals $\delta t_{n}$, and bout distances *d*_*n*_; for the aggregated multiple-fish trajectories (gray), trajectories generated from B-HMM (black), and generated from N-HMM (blue); at 22°C. **(g)** Mean Square Reorientation (MSR) accumulated after *q* bouts for aggregated multiple-fish trajectories (normal : grey, shuffled : red dashed), single-fish long trajectories (green), and trajectories generated from N-HMM (blue). For both long and N-HMM-generated trajectories, we show the mean and standard deviation over all individuals (solid line and band). (see S7 Fig for individual trajectories and all temperatures) **(h)** MSR at *q* = 10 bouts, with mean (horizontal bars) and standard deviation (vertical bars).

We report in Fig 6a, 6b, 6c the pipeline to generate 2D swim trajectories from neural recordings of individual fish. This is done by first identifying neural states from recordings using the Viterbi algorithm. We then generate a sequence of discrete bouts by sampling the behavioral distribution of inter-bout intervals $\delta t_{n}$, rescaled by the scaling factor $f_{N/B}\approx 0.44$ (see S7 Fig a for example sequences). This simulates a stochastic bout-initiation process with the correct temporal characteristics. The bout internal state *b*_*n*_ is given by the neural state identified at time $t_{n}=\sum_{n^{\prime }=1}^{n}\delta t_{n^{\prime }}$.

We then sample, for every *n*, the traveled distance *d*_*n*_ from the experimental distribution. We ignore the potential dependence of $\delta t_{n}$ and *d*_*n*_ to bout type. Lastly, we sample the emission probability $E(\delta \theta_{n}|b_{n})$ associated to the Behavioral HMM (B-HMM) inferred from all fish data to get a realization of the reorientation angle $\delta \theta_{n}$.

We reconstruct the coordinates of the virtual fish after *k* bouts, $\left(x_{k},y_{k}\right)$, through

$$x_{k}=\sum_{n=1}^{k}d_{n}\cos(\theta_{n})\;,\;y_{k}=\sum_{n=1}^{k}d_{n}\sin(\theta_{n})\;,$$ (5)

where $\theta_{n}=\sum_{n^{\prime }=1}^{n}\delta \theta_{n^{\prime }}$ is the orientation angle of the fish at bout *n*, constructed as the cumulative sum of re-orientation angles at previous bouts.

In Fig 6c, we show a representative synthetic trajectory generated from neural data with the above procedure (see S7 Fig b for more examples).

#### 2.7.2 Generation of synthetic 2D trajectories from neural model.

We can further push the N-HMM models associated to individual fish to generate *de novo* synthetic 2D trajectories, not associated to a specific neural recording. To this aim we first generate synthetic temporal sequences of neural states $s_{n}^{N}\in \{F,L,R\}$ (Fig 6d) with the N-HMM. We then use the distributions of inter-bout intervals, traveled distances, and reorientation angles to sample synthetic trajectories as explained above. An example trajectory is shown in Fig 6e.

The similarity with real trajectories is quantified by the comparison of the distributions of bout angles, inter-bout duration intervals and traveled distances. As shown in Fig 6f the distribution of bout angles, inter-bout duration intervals and traveled distances is in very good agreement with the ones observed in the behavioral data.

We further characterize the trajectories using the Mean Square Reorientation (MSR) after *q* bouts:

$$\mathrm{MSR}(q)=\langle (\theta_{n+q}-\theta_{n}{)}^{2}\rangle$$ (6)

where the average is taken across all bouts *n* and all trajectories.

Fig 6g shows the values of MSR(q) obtained from N-HMM-generated trajectories at different temperatures (see S7 Fig d for the remaining temperatures), as well as the MSR directly obtained from multiple-fish trajectories and long single-fish trajectories (only at $26^{\circ }$C).

We first notice that long individual fish trajectories at $26^{\circ }$C display large variability in MSR(q) values, compatible with the presence of fish-to-fish variability. This variability is washed out for the multiple-fish dataset (since individual trajectories are combined) providing an averaged MSR(q) for each temperature. Interestingly, the MSR for the long sequences of individual animals significantly differ from the MSR obtained from multiple-fish trajectories. This could be due to differences in experimental conditions, and in particular the effects of collective vs. isolated navigation [24].

N-HMM-generated trajectories have a MSR distribution compatible and encompassing the behavioral data in their variability. Such large variability is expected from the large fluctuations in neural brain states. Some trajectories generated with N-HMM show anomalously large angular persistence (see S7 Fig a), which may correspond to brain states where the *Anterior Rhombencephalic Turning Region* (ARTR) displays no left-right alternating behavior. This is expected, as the N-HMMs were established from spontaneous activity recordings of immobilized fish which were not constrained to swim-like behaviors. In Fig 6h, we summarized and compared the results for the Mean Square Reorientation after 10 bouts, MSR(q=10) (see S7 Fig e for all temperatures). We found, consistently across temperatures, that the MSR of behavioral data are comprised within the one-standard-deviation confidence interval of N-HMM-generated trajectories.

As we show in S1 Text, the MSR of HMM-generated trajectories can be decomposed as the sum of a purely diffusive contribution, associated to the variance of bout angles, and of terms arising from time-correlations in bout type selection along a trajectory (see Eq (12)). The increase of bout-angle variance with temperature is sufficient to explain the increasing trend of the mean MSR with temperature observed in Fig 6h (see S7 Fig f–g).

## 3 Discussion

With the advancement of video-tracking and brain recording methods, behavioral neuroscience has changed radically in the last decade. It is now possible to study in great details animal behavior in unconstrained naturalistic conditions [40–42], while new recording methods give access to extended circuit activity encompassing several brain regions. Such experiments produce vast amounts of high-dimensional data, requiring automated yet robust and interpretable analysis methods.

An essential task is the identification of behavioral or neural states from the segmentation of the recorded time series, in order to extract low-dimensional representations that are easier to interpret. However, no definitive procedure exists for selecting the optimal number of states or for defining valid labeling criteria. This choice typically depends on available observables and involves a compromise between interpretability and accuracy of representation.

In our case, the difficulty stems from three main factors: (i) the dependence of swim bout kinematics with bath temperature, (ii) the inter-individual variability, and (iii) the overlapping distributions of reorientation angles for distinct bout types, in particular at low temperatures. Because they can accommodate such overlaps while taking into account the temporal regularities in the bout sequences, Hidden Markov Models (HMMs) are ideally suited for such a task. They are easily interpretable as the dynamics between the different internal states is described by a Markovian process. This makes HMM a powerful alternative to deep-learning-based methods, whose predictive power comes at the cost of interpretability.

In this study, we successfully applied a three-state HMM to parse behavioral and neural time series associated with exploratory dynamics. We showed that for behavioral data, HMM provided a less biased and more consistent method for bout-type labeling compared to standard threshold-based Markov Chain (MC) methods used in earlier studies.

This robustness proved essential as we investigated the effect of bath temperature on navigation. Zebrafish being cold-blooded animals, the water temperature is expected to directly affect muscle efficiency, leading to a systematic increase in the amplitude of reorientation elicited by bouts as temperature rises. When using threshold-based MC methods, this may lead to a systematic but artifactual increase in the fraction of bouts labeled as turns with temperature. With HMM, this physiological effect of temperature is naturally accounted for through an adaptive adjustment of the effective threshold angle between turn and forward bouts. With this unbiased labeling, we found that the fractions of forward and turn bouts were only weakly dependent on temperature, in contrast with previously published analysis [24]. The primary effect of rising temperature is to progressively decrease bout-type persistence, i.e. the tendency of the animal to chain similar bouts. Interestingly, we found that all three bout types, and not just turns as previously reported, exhibit comparable persistence.

A natural extension of the present study would be to examine how the state transition statistics are changed by varying sensory contexts beyond the bath temperature. In particular, it has been shown that applying a stereovisual illumination gradient induces a bias in the selection of turning direction, favoring reorientations toward the brightest side [19,22,43]. Using the same analysis, one could train HMMs on both neuronal and behavioral data recorded under different illumination conditions, and assess whether the transition matrices are similarly impacted.

HMMs also demonstrated remarkable sensitivity to individual phenotypic variability. Inter- and intra-individual variability are ubiquitous traits of animal behavior [44,45] and are necessary to ensure a trade-off between flexibility and adaptability to changing environmental demands and robustness in neural development [46,47]. In [24], inter-individual differences were demonstrated on the same dataset using multiple kinematic parameters (including inter-bout interval, forward travel distance or reorientation amplitude). In contrast, our study shows that HMM can identify individual fish solely based on the dynamics of bout-type sequences. Moreover, HMM provides explicit likelihood evaluation for bout sequences for various individual-specific models, providing a quantitative measure of phenotypic proximity between animals or across time.

Since our approach is based on gait phenotyping and is independent of image features, it is compatible with low-resolution videos (in which only the animal’s position and orientation can be accessed) while still keeping versatility, reliability, and fast execution. This opens new opportunities for studying phenotypic variation in swimming behavior, potentially uncovering subtle effects on behavior of genetic, developmental, or environmental cues. This ability to precisely capture behavioral variability might also prove fruitful in order to explore the neural basis of individuality.

The fact that the fish directional dynamics can be described by a three-state Markovian sequence, suggests that bout-type selection is likely governed by a single circuit, with the ARTR being the most plausible candidate. Since its discovery in 2012 [32], the ARTR has been viewed as a direction-selection hub, controlling lateralized behaviors such as tail flick and ocular saccade orientation [20,43]. It also responds to lateralized visual stimuli, including binocular contrast and whole-field lateral motion [22,43].

In the present study, we showed that a three-state HMM can accurately describe ARTR neuronal data, and that this model is structurally and temporally similar to behaviorally-trained HMMs. This result suggests that the ARTR may also govern forward bout selection, unifying the control of all directional bout types within a single circuit. This interpretation is reinforced by the generation of synthetic, neurally driven swimming sequences that closely matched the statistics of observed trajectories. We acknowledge that this hypothesis rests only on the comparison of a behavioral and a neuronal model obtained from data recorded separately. In order to confirm it, one would need to perform experiments with joint behavioral and neuronal recordings, where swim bouts can be segmented into left/right/forward bouts. However, such a dataset is not yet available. The closest which exist are of paralysed fish where fictive behavior is inferred from electrophysiological recordings of motor neurons in the spinal cord. While those recordings provide a good estimation of motor command asymmetry [20,48], to the best of our knowledge they cannot be segmented into left/right/forward bouts.

Bout-type persistence, as observed in behavioral assays, is mirrored in the slow sequential exploration of the three hidden states identified in neural recordings of the ARTR. Although the HMM enables the identification of these neuronal states, they provide no interpretation of how they emerge from interactions among the ARTR neuronal population. In fact, our implementation of HMM assumes the activity of neurons to be independent of each other when conditioned to a state.

In a recent study, we trained data-driven graphical models (Ising model) on ARTR activity sequences [29]. The Ising model uses activity patterns to learn the interactions between neurons but, unlike HMM, it ignores any temporal information in the data. Interestingly, the inferred Ising models tend to display three metastable states, two with high activity on either side and one “equilibrated” state with intermediate, balanced activity on both sides, consistent with the three hidden states found with HMM. This convergence underlines the complementary strengths of state-space and energy-based models in elucidating neural dynamics. While the former might enable capturing the temporal structure in collective neural activity, the latter offer insights into the underlying network interactions driving these states, and how metastability emerges within neural populations. [29] also found that the metastability was explained mostly by two effective macroscopic parameters: the effective neural input on each recorded neuron and a connectivity parameter for each pair of neurons. We may speculate that the behavioral individuality differences we observed may be reflected in slight differences of such neural input or connectivity parameters, which make the mean passage times between the 3 identified metastable states slightly different.

Current evidence suggests that the decision to initiate a swim bout and the choice of its direction are mediated by partially distinct circuits. Pre-motor nuclei such as the nucleus of the medial longitudinal fasciculus (nMLF) and the mesencephalic locomotor region (MLR) act as “swim-drive” centers. The first was shown to be activated by visual stimuli inducing forward bouts [49], and the second was shown to elicit forward bouts and scale swimming speed when stimulated [50].

Turning, by contrast, relies on lateralized descending inputs. A subset of ventral spinal projection neurons (vSPNs, including RoV3, MiV1 and MiV2) which fire asymmetrically during the first tail undulation, biasing the bout toward the turning side; subsequent undulations then resemble those of forward swims. These vSPN follow a sigmoid profile, with low activity during contraversive swims, high during ipsiversive swims, and intermediate during forward swims [33], which is coherent with the left-active, right-active, and balanced states uncovered by our HMM analysis.

While the exact role of the ARTR in controlling bout selection remains unclear, anatomical tracing shows that it projects onto the vSPN domain [20], suggesting that it might drive this bias. Our data are consistent with a model in which the left and right ARTR subpopulations inhibit contraversive turns: when both sides are equally active they suppress turning, leaving forward bouts as the default. Such a motor suppression mechanism is consistent with observations by [20], who showed a monotonic relationship between the activity of ARTR neurons, and the lateralization of fictive bouts. Visually driven locomotion may bypass this mechanism via an ARTR-independent pathway, as suggested by [51], whereas spontaneous swimming could rely more heavily on ARTR-mediated bout selection.

Overall, we have found no incompatibility in the literature with our proposed model of the ARTR as a modulator of both forward and left-right swim orientations. We suggest two tests of this hypothesis. First, a detailed analysis of ARTR dynamics during forward swims, for example during forward OMR stimulation, in order to test whether its intrinsic oscillation persists or becomes phase-locked in a balanced state. Second, a bilateral ablation of all ARTR neurons to assess their necessity for spontaneous versus visually evoked locomotion, by comparing bout frequency and orientation after lesions.

Last of all, to facilitate the accessibility and adoption of Hidden Markov Model (HMM) formalism for analyzing behavioral sequences, we provide a comprehensive and instructive Python tutorial (https://github.com/EmeEmu/IBIO-Banyuls2023-Python). This tutorial can be adapted for specific datasets or used as a resource for broader educational goals.

## 4 Materials and methods

### 4.1 Behavioral datasets

The behavioral dataset used in the present study is derived from [24], and can be accessed directly at https://doi.org/10.5061/dryad.3r2280ggw. This dataset comprises spontaneous swimming trajectories of 5 to 7 dpf zebrafish larvae, collected at controlled bath temperatures of 18°C, 22°C, 26°C, 30°C, and 33°C. A camera was used to continuously record the swimming behavior of the fish within an arena of 100×45×4.5mm^3^ for 30 minutes at 25 frames/second. To eliminate border effects, a Region of Interest (ROI) was defined at a distance of 5mm from the arena’s walls. Fish that swam outside the defined tracking ROI were considered lost, and a new trajectory was initiated upon their re-entry into the ROI. The identity of the fish is thus lost each time it exits the ROI. Therefore, the dataset contains a varying number of fish trajectories, ranging from 532 to 1513 trajectories across the different temperatures (mean = 1148). Individual trajectories were tracked offline using the open-source FastTrack software [52], and were then discretized into sequences of swimming bouts.

Each trajectory consists of a sequence of swim bouts, spanning from 9 to 748 bouts per trajectory (mean=60, distributions shown in S1 Fig a). From this extensive dataset, we primarily utilized the re-orientation angles, defined as the difference between the heading direction at bout *n* + 1 and the heading direction at bout *n*:

$$\delta \theta_{n}=\theta_{n+1}-\theta_{n}$$ (7)

(a graphical illustration of this definition can be found in Fig 1c). This parameter encapsulates the angular change between consecutive bouts, providing insight into the fish’s ability to modify its orientation during swimming.

We also used the interbout interval $\delta t_{n}=t_{n+1}\;-\;t_{n}$ representing the elapsed time between 2 consecutive bouts, and the traveled distance $d_{n}=\|{\vec{r}}_{n+1}-{\vec{r}}_{n}\|$.

On top of these multi-fish trajectories, we used in Sects 2.4 and 2.7 a second dataset from [24] consisting in single-fish recordings. For this dataset, each fish (N = 18) is placed alone in the arena at 26°C, and is recorded for 2 hours. With this experimental paradigm, the identity of the fish is conserved across trajectories, even when the fish leaves and re-enters the ROI.

### 4.2 Neuronal datasets

The neuronal dataset used in the present study is derived from [29], and can be accessed directly at https://gin.g-node.org/Debregeas/ZF_ARTR_thermo. This dataset contains 32 one-photon Light-Sheet Microscopy recordings of spontaneous brain activity, for 13 zebrafish larvae (5 to 7 dpf) at 18°C, 22°C, 26°C, 30°C, and 33°C. It focuses on neurons from the *Anterior Rhombencephalic Turning Region* (ARTR), with ∼300 neurons (mean 307, std 119), recorded during ∼20min (mean 23, std 4 min) at ∼6 Hz (mean 5.9, std 2.1 Hz). The approximate binarized spike trains of segmented neurons were inferred from fluorescence signals using a previously described deconvolution algorithm [53].

### 4.3 Emission of reorientation angles in the Hidden Markov model

To validate the hypothesis that the re-orientation angles can be modeled using normal and gamma distributions, we compared the distribution of the data with a Gaussian Mixture Model (GMM) and a Gaussian & Gamma Mixture Model:


$$p(\delta \theta )=w_{F}𝒩(\delta \theta ;0,\sigma )+w_{L}\Gamma (\delta \theta ;\alpha ,\theta )+w_{R}\Gamma (-\delta \theta ;\alpha ,\theta )$$

where $w_{F}+w_{L}+w_{R}=1$, and *w*_*F*_, *w*_*L*_, and *w*_*R*_ denote the weights for forward, left, and right states, respectively.

Using Quantile-Quantile (QQ) plots, we show that this last mixture model accurately reproduces the observed distribution of $\delta \theta_{n}$ in the data, and is much better than a GMM, especially in the tails of the distributions (S2 Fig a). We also confirmed that, once trained, the emission distributions do indeed match the observed reorientation distributions (S2 Fig b–c).

### 4.4 Hidden Markov model training

Given a data set of trajectories (neuronal or behavioral), the Hidden Markov models were trained using the standard Baum-Welch algorithm [54]. We train for a maximum of 500 expectation-maximization iterations, stopping earlier if the gain in the total log-likelihood of all the data in one iteration becomes less than a small threshold, that we fix at 10^−6^. In practice we find that all HMMs trained in this study converge before reaching 500 iterations.

The transition matrix parameters are initialized at random, respecting left-right symmetry in the case of the behavioral models.

### 4.5 Stubbornness factor

The *stubbornness* factor *f*_*q*_ is a measurement of the animal’s preference towards turning in the same direction over changing direction, after *q* intermediary forward bouts (S4 Fig c), as defined in Eq (3).

It can be computed from a sequence of classified bouts *b*_*n*_ by first identifying and counting the q-plets $T_{1}\to F^{q}\to T_{2}$ where $T_{1}=T_{2}$ and where $T_{1}\neq T_{2}$:

$$\left\{ \begin{array}{cc} N_{=} & =\#(T_{1}\to F^{q}\to T_{2},T_{1}=T_{2})\\ N_{\neq } & =\#(T_{1}\to F^{q}\to T_{2},T_{1}\neq T_{2}) \end{array}\right.$$ (8)

and then computing their ratio:

$$f_{q}=\frac{N_{=}}{N_{\neq }}$$ (9)

In practice, this ratio has a physical interpretation only for long sequences of bouts where *N*_=_ >>1 and $N_{\neq }>>1$. As the trajectories in our dataset can be quite short (S1 Fig a), we compute *f*_*q*_ from all trajectories at a specific temperature, increasing the chance of observing a high number of stubborn (*N*_=_) and non-stubborn ($N_{\neq }$) trajectories.

By considering that the probability of a given q-plet is stubborn follows a binomial distribution ($𝔼(N_{=})=pN$ and $𝔼(N_{\neq })=(1-p)N$ with $N=N_{=}+N_{\neq }$), we can evaluate the uncertainty in *stubbornness* as:

$$\Delta f_{q}=f_{q}\frac{1}{N_{=}+N_{\neq }}(\sqrt{\frac{N_{=}}{N_{\neq }}}+\sqrt{\frac{N_{\neq }}{N_{=}}})$$ (10)

It is to be noted that these uncertainties are conservative estimates, as there exits a bias inherent to the dataset. Indeed, a very stubborn fish will tend to stay longer within the Region Of Interest (ROI) of the camera, leading to longer trajectories and therefore weighing more on the final result. Hence, it is unclear whether a *stubbornness* factor $f_{q}=1\pm 0.2$ is truly significant (as suggested by the estimated error bars on S4 Fig d).

Furthermore, as the stubbornness factor is computed from all trajectories (and thus all fish) at a particular temperature, it represents an average behavior rather than an individual fish.

### 4.6 Stubbornness factor and 3-state Markov Chain

The *stubbornness* factor can be defined directly from the transition matrix:

**For *q* = 0,** calculations are simple:

$$f_{q=0}=\frac{P(L\to L)+P(R\to R)}{P(L\to R)+P(R\to L)}$$ (11)

**For q≥1,** the *stubbornness* factor is defined from the transition matrix as:


$$S_{L,q}=P(L\to F^{q}\to L)$$

$$=P(L)P(L\to F)P^{q}(F\to F)P(F\to L)$$

$$W_{L,q}=P(L\to F^{q}\to R)$$

$$=P(L)P(L\to F)P^{q}(F\to F)P(F\to R)$$

$$f_{q}=\frac{S_{L,q}+S_{R,q}}{W_{L,q}+W_{R,q}}$$

with *S*_*L*,*q*_ the probability of a trajectory which starts and ends with a left bout, *W*_*L*,*q*_ the probability of a trajectory which starts with a left bout and ends with a right bout, and *S*_*R*,*q*_ *W*_*R*,*q*_ their symmetrical opposites.

For a 3-state model, the forward-forward bout probability cancels out, giving:


$$f_{q}=\frac{P(L)P(L\to F)P(F\to L)}{P(L)P(L\to F)P(F\to R)}$$

$$\frac{+P(R)P(R\to F)P(F\to R)}{+P(R)P(R\to F)P(F\to L)}$$

and with our non-handedness hypothesis: P(L)=P(R), P(L→F)=P(R→F), and P(F→L)=P(F→R), yielding:

$$f_{q}=1\;\forall q>0$$ (12)

### 4.7 Labeling of states in the neuronal Hidden Markov Model

The internal states of the Hidden Markov Models (HMMs) trained from neuronal activity are not *a priory* assigned to the Left, Right and Forward labels, and must therefore be re-ordered post-training.

We expect a certain symmetry in the system, where neurons in the left side of the ARTR will be more active during a Left state (and vice versa). Hence, we can use the excitability $h_{i}^{s}$ of neuron *i* in each internal state *s*, as defined in the emission distribution of the HMM (see Eq 4). We define the lateralized excitability:

$$\Delta h^{s}=\langle l(h_{i}^{s})\rangle_{i\in 𝔏}-\langle l(h_{i}^{s})\rangle_{i\in ℜ}$$ (13)

where $l(x)=\frac{1}{1+e^{-x}}$ is the standard logistic function, and 𝔏 and ℜ are the sets of neurons located respectively in the left and right side of the ARTR. We thus label the HMM states such that

$$\Delta h^{L}>\Delta h^{F}>\Delta h^{R}$$ (14)

with *F*, *L*, and *R* the Forward, Left and Right internal states.

### 4.8 Temporal re-scaling

To find the temporal re-scaling factor *f*_*N*/*B*_ between behavioral and neuronal models, we first compute the distributions of sojourn times $\Delta t_{s}$ for all states s∈{F,L,R} in both behavioral and neuronal Hidden Markov Models.

We then find the optimal re-scaling factor *f*_*N*/*B*_ for which the combined distributions $\Delta t_{b}=[\Delta t_{F}^{b},\Delta t_{L}^{b},\Delta t_{R}^{b}]$ and $\Delta t_{n}=[\Delta t_{F}^{n},\Delta t_{L}^{n},\Delta t_{R}^{n}]$ are as close to each other as possible :

$$f_{N/B}=\min_{f\in [0,1]}\text{RMSE}(Q(\Delta t_{b}),f.Q(\Delta t_{n}))$$ (15)

where *Q*(*D*) is the quantiles of a distribution *D*, and RMSE(𝐱,𝐲) is the Root Mean Squared Error between vectors **x** and **y** (see S6 Figa).

For Markov chains, the transition matrix $P=P(s_{n}=s\to s_{n+1}=s^{\prime })$ represents the probability of transitioning in one step from state *s* to state $s^{\prime }$. The transition probability $s\to s^{\prime }$ in $k\in {\mathbb{N}}_{1}$ steps $P(s_{n}=s\to s_{n+k}=s^{\prime })$ is then the matrix power *P^k^*.

In order to apply the temporal re-scaling *f*_*N*/*B*_ between behavioral and neuronal models, we can thus compute the re-scaled transition matrix :

$$P_{n}^{*}=P_{n}^{\left\lfloor \frac{\nu }{f_{N/B}}\right\rceil }$$ (16)

where *P*_*n*_ is the transition matrix inferred from neuronal data recorded at a frequency ν Hz.

### 4.9 Mean square reorientation

To characterize the orientational diffusivity of the trajectories, we use the Mean Square Reorientation (MSR) accumulated after *q* bouts, as defined in Eq (6) [22].

For infinitely large datasets with no left-right bias, we expect a centered distribution of reorientation angles $\langle \delta \theta_{n}\rangle_{n}=0$. However, this is not the case, particularly for the neuronal dataset where experimental limitations can induce strong biases. In particular, two of those limitations are due to the one-sided illumination in our Light Sheet Fluorescence Microscope [55]. First, due to scattering, the illumination beam is not uniform left-right across the brain, which can induce biases in the detection of neurons and their activity. Second, the non-uniform perception of light by the zebrafish larvae can elicit a phototaxis response, which is known to bias the activity of the *Anterior Rhombencephalic Turning Region* (ARTR) [43].

Since a non-zero bias can result in a distortion of the MSR (see S1 Text), the MSR is computed from $\hat{\delta \theta_{n}}=\delta \theta_{n}-\langle \delta \theta_{n}\rangle_{n}$ instead of $\delta \theta_{n}$.

## Supporting information

S1 Fig Supplementary panels to Fig 1. **(a)** Number of bouts per trajectory for the entire behavioral dataset (black), and per temperature (inset, colored). **(b)** Observed transition probabilities between reorientation angles for the entire behavioral dataset. **(c)** Difference between mean activities in the left (*m*_*L*_) and right (*m*_*R*_) *Anterior Rhombencephalic Turning Region* for all fish at each temperature.
(EPS)

S2 FigSupplementary panels to Fig 2 - Emission distributions.**(a)** Quantile-Quantile plot between the empirical distributions of reorientation angles and two Mixture Models. *Left:* Mixture Model defined from a central Normal distribution (forward bouts) and two Gamma distributions (left and right turning bouts), corresponding to the model of HMM emissions. *Right:* Gaussian Mixture Model. *Insets:* Zoom on $\pm 50^{\circ }$. **(b)** Quantile-Quantile plot between the empirical distributions of reorientation angles and the distributions of reorientation angles generated by HMM. *Insets:* Zoom on $\pm 50^{\circ }$. **(c)** Empirical distributions of reorientation angles (colored) vs. distributions generated by the 3-state Hidden Markov Model (HMM; black), at each temperature. **(d)** Distributions of absolute reorientation angles labeled as forward bouts (solid black) and turning bouts (left or right; solid pink) by the Hidden Markov Model (HMM). Dashed lines show the HMM emission distribution for forward and turning bouts (black and pink respectively). The vertical black line shows the threshold $\delta \theta_{0}=10^{\circ }$ used in the Markov Chain model. **(e)** HMM emission parameters : σ the standard deviation of the central Normal distribution (forward state), α and θ the shape and scale of the Gamma distribution (turning states). Each dot corresponds to one temperature, and error bars were computed from the minimum-maximum of 100 cross-validations (trained on randomly selected 50% of the datasets).(EPS)

S3 FigSupplementary panels to Fig 2 - Comparison between Markov Chain and Hidden Markov Model.**(a)** 3-state Markov Model (HMM) with a threshold at $\pm \delta \theta_{0}=10^{\circ }$ to classify reorientation angles δθ into forward/left/right states. **(b)** Steady state bout probabilities *P*(*s*) vs. temperature, for forward (*s* = *F*, black) and turning bouts (s∈L,R, pink), and for both Markov Chain inferred from threshold (MC, circles) and Hidden Markov Models (HMM, triangles). **(c)** Transition probabilities $P(s_{n}\to s_{n+1})$ vs. temperature, for both Markov Chain (MC, circles) and Hidden Markov Model (HMM, triangles). **(b,c)** Shaded curves represent the minimum-maximum of 100 cross-validations of both models inferred from randomly selecting 50% of the data. **(d)** Transition matrices between forward (F), left (L) and right (R) states, for both the Markov chains inferred from thresholded data (MC, top) and Hidden Markov Model (HMM, bottom), at each temperature. **(e)** Confusion matrices between labeling of MC and HMM at each temperatures (normalized with respect to MC labeling).(EPS)

S4 FigSupplementary panels to Fig 2 - Markovianity.**(a)** Distribution of log Likelihoods (LLHs) for both the Markov chains inferred from thresholded data (left) and Hidden Markov Model (right). For each model and each temperature, LLHs were computed for 100 models inferred from 50% of the trajectories (randomly constructed training set) and on the remaining 50% of the trajectories (testing set). Dashed lines show the quartiles of each distribution. **(b)** 4-state Markov chain used in previous publications [22,24]. Two Markov Chains run in parallel, with the first chain controlling bout type (forward or turn) and the second controlling direction (left or right). The system can be in one of four states: [*T*,*L*],[*T*,*R*],[*F*,*L*],[*F*,*R*], thus left and right states represent internal directional states (not just observed behavioral orientations). **(c)** Graphical definition of the stubbornness. For a q-plet of bouts $T_{1}\to F\to \ldots \to F\to T_{2}$ with *q* intermediary forward bouts, a stubborn sequence is defined as one where directionality is conserved (i.e. $T_{1}=T_{2}$), whilst a non-stubborn sequence will lose the memory of the initial turn (i.e. $T_{1}\neq T_{2}$). **(d)** Stubbornness factor *f*_*q*_ (see Eq 3) vs. number of intermediary forward bouts *q*, for both Markov Chain inferred from thresholded trajectories (MC, dots) and the Hidden Markov Model (HMM, triangles) trained directly from reorientation angles, at each temperature. The width of the shaded curves represent the estimated error in *stubbornness* factor (see Materials and Methods 4.5).(EPS)

S5 Fig Supplementary panels to Fig 3. Hidden Markov Model parameters inferred from all trajectories from an individual fish, compared with the average parameters inferred from chunks of that fish’s trajectories. All HMM parameters are shown. Each dot represents a fish, with error bars corresponding to standard error of the mean. Blue color corresponds to real individual fish data. Gray points are obtained by sampling long trajectories from a single HMM trained on all fish bundled together, thus representing a null model for the fish individuality.
(EPS)

S6 FigSupplementary panels to Fig 5.**(a)** Root Mean Squared Error (RMSE) between quantiles of the behavior and neuronal sojourn distributions presented in Fig 5a at different values of the rescaling factor *f*. The optimal rescaling factor corresponds the minimal RMSE at $f=f_{N/B}\approx 0.44$. **(b)** Transition probabilities $P(s\to s^{\prime })$ between hidden states *F*, *L*, and *R*, for the behavioral HMM (black), and neuronal HMMs before (orange) and after rescaling by *f*_*N*/*B*_ = 0.44 (magenta), at each temperatures. Shaded curves represent the standard error of the mean over all fish.(EPS)

S7 Fig Supplementary panels to Fig 6. **(a)** Example empirical ARTR activity at 26°C (top) and multiple corresponding state sequences after temporal re-scaling and bout sampling (bottom). **(b)** Reconstructed trajectories from the empirical neuronal activity presented in panel a (for each sampled state sequence). **(c)** Example B-HMM-generated trajectory at 26°C (color = bout type (left, right, forward), point size = inter-bout interval). **(d)** Mean Square Reorientation (MSR) after *q* bouts from aggregated multiple-fish trajectories at 18-33 °C (grey), long-individual trajectories at 26° C (green) and trajectories generated from Neural HMM (N-HMM, blue). Red dashed lines are MSR obtained from shuffled aggregated multiple-fish trajectories. **(e)** MSR (q = 10) for data and N-HMM-generated trajectories, with mean (horizontal bars) and standard deviations (vertical bars). **(f-g)** Same as panels d-e but for the standardized MSR where trajectories are normalized such that the bout angles have unit variance. See Eq (12).
(EPS)

S8 FigCross-validation of 3-state HMM for Neural ARTR data.**A)** Correlation between two-point averages $\left\langle \sigma_{i}\sigma_{j}\right\rangle$ estimated by the HMM and their empirical counterparts, as a function of the number of hidden states in the HMM. This correlation is computed for each temperature, and the plot shows only the average and the standard deviation. **B)** Average log-likelihood per unit time of withheld data as a function of the number of hidden states in the HMM. This correlation is computed for each temperature, and the plot shows only the average and the standard deviation. **C)** Comparison of correlation of the HMM two-point averages to their empirical counterparts for each temperature, in the 2-state (x-axis) vs. the 3-state models (y-axis). **D)** Same as C), but comparing the log-likelihood per unit time. **E)** Same as C), but comparing 3-state HMM to 4-state HMM. **F)** Same as D), but comparing 3-state HMM to 4-state HMM.(EPS)

S9 FigComparion of transition probabilities inferred with unconstrained and symmetric behavioral HMMs.We trained behavioral HMMs as defined in the main text on the first dataset. The figures compares the nine inferred transition probabilities $P(s\to s^{\prime })$ (y-axis) defining the unconstrained HMM with their counterparts in the symmetric HMMs, in which Left-Right symmetry is imposed in the transition matrix. The excellent agreement confirms that the models spontaneously learn symmetric transitions. In the main text, we impose Left-Right symmetry to speed up training and improve accuracy. See also S10 Fig. In both cases the emission distributions for left and right bouts are the same.(EPS)

S10 Fig Comparison of transition probabilities inferred with unconstrained and symmetric behavioral HMMs on long trajectories. Same as S9 Fig, but for long trajectories of individual fish. Different colors correspond to different fish.
(EPS)

S11 FigHistogram of empirical $\left(P(x_{t}|x_{t-1},x_{t-2})\;-\;P(x_{t}|x_{t-1}))/P(x_{t}|x_{t-1}\right)$ across experimental trajectories, where $x_{t}$ are states (F, L, R) determined by thresholding bout angles at $10^{\circ }$.Actual data is shown in blue. For comparison, we generated fictitious trajectories from a Markov model with transition probabilities $P(x_{t}|x_{t-1})$ calculated from the empirical transition frequencies. The corresponding histogram is shown in yellow.(EPS)

S1 Text Mean squared reorientation. (PDF)
