# Supplementary material for: Linking brain and behavior states in Zebrafish Larvae locomotion using hidden Markov models
Source: PLoS Comput Biol. 2026 Jan 5;22(1):e1013762. doi: 10.1371/journal.pcbi.1013762 (PMC12803470; doi:10.1371/journal.pcbi.1013762)
Supplement: S1 Text — (PDF) [file pcbi.1013762.s012.pdf]

Supporting Information

Linking Brain and Behavior States in Zebrafish Larvae Locomotion using Hidden Markov Models

MEAN SQUARED REORIENTATION

The mean-square reorientation (MSR) of a trajectory at lag q is defined as [1]:

$$M_q = \mathrm{E}\left[\left(\sum_{i=1}^{q} \delta\theta_{t+i-1}\right)^2\right] = \sum_{i,j=1}^{q} \mathrm{E}\left[\delta\theta_{t+i-1}\delta\theta_{t+j-1}\right] \tag{1}$$

where it is assumed that $\mathrm{E}[\delta\theta] = 0$. The average is taken over time t. Assuming stationarity, this is independent of t, and should thus only depend on the separation $|i-j|$,

$$\mathrm{E}\left[\delta\theta_{t+i-1}\delta\theta_{t+j-1}\right] = \mathrm{E}\left[\delta\theta_i\delta\theta_j\right] = A_{|i-j|} \tag{2}$$

where $A_{|i-j|}$ stands for the time equilibrated autocorrelation function:

$$A_t = \lim_{t_0\to\infty} \mathrm{E}[\delta\theta_{t_0}\delta\theta_{t_0+t}] \tag{3}$$

In particular $A_0 = \mathrm{E}[\delta\theta^2]$ is just the variance of $\delta\theta$. It follows that,

$$\begin{aligned} M_q &= \sum_{i,j=1}^{q} A_{|i-j|} = \sum_{t=0}^{q-1}\left(\sum_{i,j=1}^{q} \delta_{|i-j|,t}\right) A_t \\ &= qA_0 + 2\sum_{t=1}^{q-1}\left(\sum_{i<j}^{q} \delta_{j-i,t}\right) A_t \\ &= qA_0 + 2\sum_{t=1}^{q-1}(q-t)A_t \end{aligned} \tag{4}$$

Note that for a random walk without any correlations across time, $A_t = 0$ for $t > 0$. In this case, $M_q = qA_0$ grows linearly with q.

On the other hand, it is expected that $A_t \to 0$ as $t \to \infty$, and usually this decay is exponentially fast in time. Therefore, for large q, we get the following asymptotic expression for M_q:

$$M_q \sim \left(A_0 + 2\sum_{t=1}^{\infty} A_t\right) q - 2\sum_{t=1}^{\infty} tA_t \tag{5}$$

Notice that this is affine in q, with the coefficient $A_0 + 2\sum_{t=1}^{\infty} A_t$. Therefore, M_q is initially linear in q with slope A_0 for small q, then has an elbow and eventually approaches the asymptotic slope $A_0 + 2\sum_{t=1}^{\infty} A_t$ as $q \to \infty$. This asymptotic slope is different from A_0 only if the process exhibits non-trivial autocorrelations in time.

A. MSR for the HMM

As an illustration, we can compute all these quantities exactly for the HMM as follows. For the autocorrelation, we have:

$$\begin{aligned} A_t &= \operatorname*{tr}_{h_0,\dots,h_t} P(h_t|h_{t-1})\dots P(h_2|h_1)P(h_1|h_0)P(h_0) \\ &\quad\times\left[\int P(\delta\theta|h_0)d\delta\theta\right]\left[\int P(\delta\theta|h_t)d\delta\theta\right] \\ &= \operatorname*{tr}_{h,h'}[\Omega^t]_{h',h}P(h)\langle\delta\theta|h\rangle\langle\delta\theta|h'\rangle \end{aligned} \tag{6}$$

where $[\Omega]_{h',h} = P(h'|h)$ is the transition matrix of the HMM. We will assume here that the initial state is sampled from $P(h) = p_{\rm eq}(h)$, the equilibrium distribution of hidden states of the HMM, which satisfies the stationarity equation:

$$\mathop{\rm tr}_h \Omega_{h',h} p_{\rm eq}(h) = p_{\rm eq}(h') \tag{7}$$

Note also that $\mathrm{E}[\delta\theta] = 0$ implies that $\sum_h p_{\rm eq}(h)\langle\delta\theta|h\rangle = 0$. Now let $p_1(h), \dots, p_L(h)$ denote the remaining eigenvectors of Ω, with the associated eigenvalues $\lambda_1, \dots, \lambda_L$. By the Perron-Frobenious theorem, these remaining eigenvalues are all smaller than one in absolute value. The vector $P(h)\langle\delta\theta|h\rangle$ can be writen in the basis of this eigenvectors,

$$P(h)\langle\delta\theta|h\rangle = \alpha_{\rm eq} p_{\rm eq}(h) + \sum_{i=1}^{L} \alpha_i p_i(h) \tag{8}$$

for some coefficients $\alpha_{\rm eq}, \alpha_1, \dots, \alpha_L$. Then it follows that,

$$\begin{aligned} A_t &= \mathop{\rm tr}_{h'} \left[\alpha_{\rm eq} p_{\rm eq}(h') + \sum_i \lambda_i^t \alpha_i p_i(h')\right] \langle\delta\theta|h'\rangle \\ &= \sum_i \alpha_i \lambda_i^t \mathop{\rm tr}_{h'} p_i(h')\langle\delta\theta|h'\rangle \end{aligned} \tag{9}$$

Since the $|\lambda_i| < 1$ it follows that $A_t \to 0$ exponentially fast as $t \to \infty$. Moreover we can compute,

$$\sum_{t=0}^{\infty} A_t = \sum_i \frac{\alpha_i}{1-\lambda_i} T_i, \quad \sum_{t=0}^{\infty} t A_t = \sum_i \frac{\alpha_i \lambda_i}{(1-\lambda_i)^2} T_i \tag{10}$$

where

$$T_i = \mathop{\rm tr}_h p_i(h)\langle\delta\theta|h\rangle \tag{11}$$

These expressions then give a complete and exact characterization of the MSR for the HMM.

B. Standardized MSR

The MSR as defined in Eq. 1 includes both the diffusive contribution from the initial term A_0 and contributions arising from non-trivial time correlations in the process coming from the terms A_t for $t > 0$. As already pointed out, this initial term $A_0 = \mathrm{E}[\delta\theta^2]$ is just the variance of the distribution of bout angles and is insensitive to time correlations. To emphasize the time correlations we may normalize the trajectories by defining:

$$\hat{M}_q = \frac{M_q}{A_0} \tag{12}$$

By comparing with Eq. 5, we see that $\hat{M}_q$ has initially a slope ≈ 1 for small q, then has an elbow and eventually approaches the asymptotic slope $1 + 2\sum_{t=1}^{\infty} A_t/A_0$ for large q.

In contrast to M_q, the quantity $\hat{M}_q$ is better suited to compare the time correlations of very diverse trajectories because it is insensitive to variations of $\mathrm{E}[\delta\theta^2]$. Figure S7 Fig c-d plots the normalized MSR from Eq. 12 for the various trajectories and temperatures considered before in Figure S7 Fig c-d. We observe that the standardized MSR exhibits comparable behavior across various temperatures, suggesting that the trend of the unnormalized MSR observed in Fig 6 b-c and S7 Fig a-b is just due to an increase in the bout angle amplitudes $\mathrm{E}[\delta\theta^2]$ with temperature, but not due to changes in the structure of their time correlations.

[1] S. Karpenko, S. Wolf, J. Lafaye, G. Le Goc, T. Panier, V. Bormuth, R. Candelier, and G. Debrégeas, From behavior to circuit modeling of light-seeking navigation in zebrafish larvae, eLife **9**, e52882 (2020), publisher: eLife Sciences Publications, Ltd.
